# Successful Stepwise Endoscopic Ultrasound‐Guided Cyst Drainage for a Giant Infected Hepatic Cyst: A Case Report

**DOI:** 10.1002/deo2.70286

**Published:** 2026-01-19

**Authors:** Kazuki Endo, Haruo Miwa, Shotaro Tsunoda, Akihiro Funaoka, Ritsuko Oishi, Yuichi Suzuki, Yusuke Takeshita, Tomoaki Takahashi, Manabu Morimoto, Shin Maeda

**Affiliations:** ^1^ Gastroenterological Center Yokohama City University Medical Center Kanagawa Japan; ^2^ Department of Gastroenterology Yokohama City University Graduate School of Medicine Kanagawa Japan

**Keywords:** cholangitis, hepatic cyst, infection control, plastic stent, ultrasound‐guided cyst drainage

## Abstract

A 79‐year‐old man presented with fever and jaundice. Laboratory tests revealed elevated inflammatory markers and hepatobiliary enzymes. Magnetic resonance imaging revealed a 20 × 16 cm giant hepatic cyst compressing the intrahepatic bile ducts. Emergency endoscopic retrograde cholangiopancreatography revealed intrahepatic bile duct dilatation secondary to cystic compression. An endoscopic nasobiliary drainage tube was inserted. After the cholangitis improved, the tube was replaced with a plastic stent. The patient was discharged but was readmitted 11 days later with recurrent fever and loss of appetite. Computed tomography revealed thickening of the cyst wall and internal debris, consistent with an infected hepatic cyst. Given the patient's poor general condition and presence of compressed vessels and bile ducts along the percutaneous puncture route, endoscopic ultrasound‐guided cyst drainage (EUS‐CD) with nasocystic drainage was performed. After clinical improvement, surgical fenestration was attempted but aborted due to inflammation and friability with bleeding around the endosonographically/EUS‐guided created route (ESCR). On day 25 after EUS‐CD, conversion to internal trans‐ESCR drainage was performed using a 7‐Fr, 15‐cm double‐pigtail stent, and the transpapillary stent was removed because bile duct compression had resolved. The infection recurred 22 days later owing to stent occlusion, requiring stent exchange and additional drainage via ESCR. Finally, three plastic stents were placed, and the patient had no further infection recurrence. After infection control with nasocystic drainage using EUS‐CD, multiple stent placements via ESCR can provide safe, effective, and durable treatment for giant infected hepatic cysts that are unsuitable for percutaneous drainage or surgery.

## 1 Introduction

Hepatic cysts are usually asymptomatic but may occasionally develop infection or intracystic hemorrhages. Although percutaneous drainage (PCD) is the standard treatment, it becomes technically challenging when the cyst is large and located near the hepatic hilum, where it compresses the surrounding vessels or bile ducts [1]. Moreover, surgical intervention is often unsuitable for patients with a poor general condition. Endoscopic ultrasonography‐guided cyst drainage (EUS‐CD) has recently been reported as a minimally invasive alternative to surgery [1, 2, 3, 4, 5, 6, 7].

Herein, we report a rare case of a giant infected hepatic cyst treated with EUS‐CD.

## 2 Case Report

**FIGURE 1:**
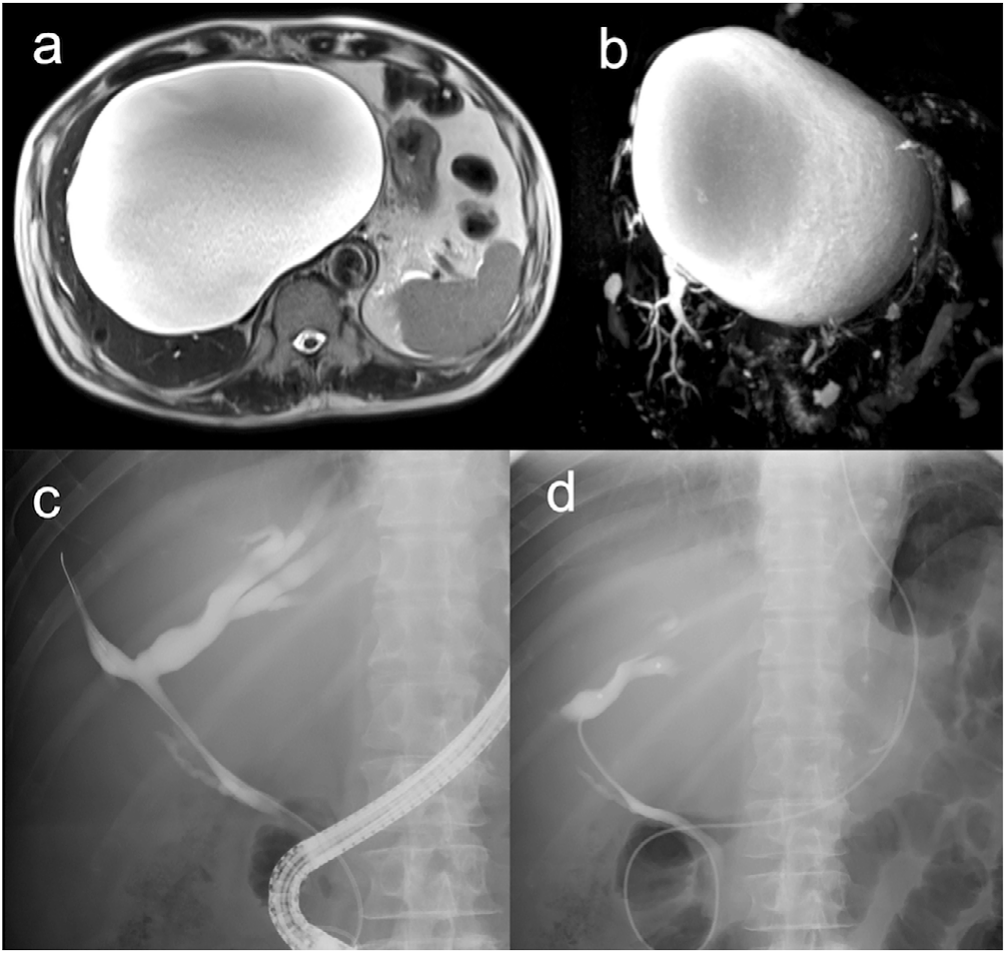
Magnetic resonance and endoscopic retrograde cholangiopancreatography (ERCP) imaging of the giant hepatic cyst and bile ducts. (a) T2‐weighted magnetic resonance imaging (MRI) shows a 20 × 16 cm hepatic cyst mainly in the left lobe, extending into the right lobe. (b) MRCP demonstrates compression of the bile duct by the cyst, with dilation of the intrahepatic bile ducts. (c) ERCP image shows dilatation of the intrahepatic bile ducts due to extrinsic compression by the giant hepatic cyst. (d) ERCP image shows an endoscopic nasobiliary drainage tube in place.

**FIGURE 2:**
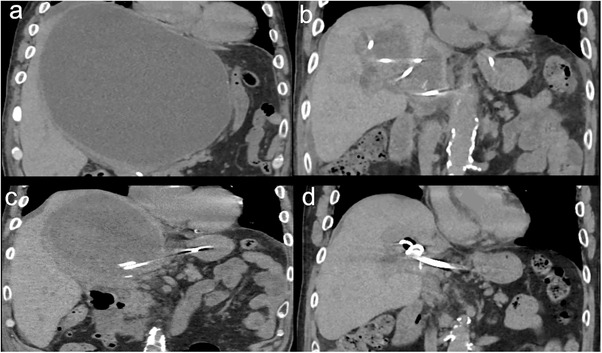
Sequential computed tomography (CT) findings of the infected hepatic cyst. (a) On readmission, the giant hepatic cyst shows wall thickening and internal hyperdensity, consistent with infection. (b) After external drainage, the cyst collapsed, increasing the distance between the cyst and the gastric wall. (c) Twenty‐two days after internal stenting, recurrence occurred due to stent occlusion. (d) Six months after placement of three internal plastic stents, the cyst remained collapsed without recurrence.

**FIGURE 3:**
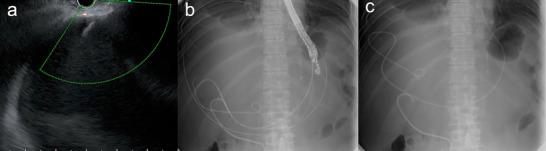
Transgastric endoscopic ultrasound‐guided cyst drainage for hepatic cyst. (a) The hepatic cyst was directly punctured from the gastric lumen under endoscopic ultrasound guidance. (b) After adequate placement of the guidewire within the giant hepatic cyst, an endoscopic nasobiliary drainage tube was successfully inserted. (c) An endoscopic nasobiliary drainage tube adequately placed within the hepatic cyst and a transpapillary biliary stent.

**FIGURE 4:**
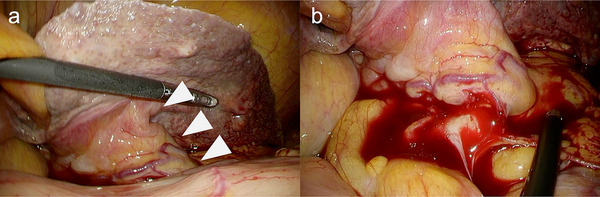
Laparoscopic view of the endosonographically/EUS‐guided created route (ESCR). (a) Laparoscopic findings show the ESCR from the stomach to the hepatic cyst (white arrowhead). (b) Active bleeding was observed from the ESCR.

A 79‐year‐old man presented with a fever and jaundice. Laboratory tests showed a white blood cell count of 9,500/µL, C‐reactive protein level of 7.3 mg/dL, total bilirubin level of 19.5 mg/dL, and elevated liver enzyme levels. Magnetic resonance imaging revealed a 20 × 16 cm hepatic cyst, mainly in the left lobe, extending into the right lobe (Figure 1a). The intrahepatic bile ducts were dilated because of cystic compression (Figure 1b), resulting in acute cholangitis. Emergency endoscopic retrograde cholangiopancreatography (ERCP) demonstrated smooth extrinsic compression at the hepatic hilum. An endoscopic nasobiliary drainage (ENBD) tube was placed in the left hepatic duct (Figure 1c,d). Cholangitis improved with biliary drainage and antibiotic administration. On hospital day 7, ERCP was repeated, and the ENBD tube was replaced with a 7‐Fr half‐pigtail plastic stent (Through & Pass Double Pit; Gadelius Medical KK, Tokyo, Japan). The patient was discharged with treatment for a giant liver cyst scheduled for 2 weeks later, but was readmitted 11 days after discharge owing to loss of appetite and general fatigue. Laboratory tests demonstrated elevated inflammatory markers, whereas hepatobiliary enzyme levels remained stable. Computed tomography revealed mild cyst wall thickening and increased internal attenuation, indicating cyst infection (Figure 2a). Blood cultures obtained on admission revealed *Klebsiella pneumoniae*. Because the patient was in poor general condition, percutaneous or endoscopic drainage was chosen as the initial treatment. However, due to marked compression of the blood vessels and intrahepatic bile ducts by the giant cyst, percutaneous transhepatic drainage was considered high‐risk. Therefore, EUS‐CD was performed. The cyst was directly punctured from the stomach using a 19‐gauge needle (EZ Shot 3 Plus; Olympus Medical Systems, Tokyo, Japan), and a 0.025‐inch guide wire (VisiGlide 2; Olympus Medical Systems) was advanced into the cyst. A 7‐Fr external nasocystic drainage tube (Flexima ENBD Catheter; Boston Scientific, Marlborough, MA, USA) was placed without dilation of the endosonographically/EUS‐guided created route (ESCR) and without the double‐guide wire technique. After the procedure, purulent fluid was drained (Figure 3). The cystic fluid was highly viscous, leading to recurrent obstruction of the drainage tube. Nevertheless, daily cyst irrigation combined with antibiotic therapy improved the inflammatory conditions. Internalization of the nasocystic drainage tube was considered; however, because the maturity of the ESCR was uncertain, the cyst had collapsed, and the enlarged distance between the cyst and gastric wall increased the risk of stent migration (Figure 2b). As external drainage requires daily irrigation to prevent occlusion, surgical fenestration was attempted to achieve durable decompression and prevent recurrence. At surgery, the cyst had collapsed, and the cyst wall was thickened. In addition, the cyst wall and area around the ESCR were severely inflamed and prone to bleeding. Therefore, wide fenestration with electrocautery was considered unsafe because of the high risk of hemorrhage, and the procedure was aborted (Figure 4). The procedure was completed using peritoneal lavage and drainage. Conversion to an internal stent was performed 25 days after EUS‐CD, and a 7‐Fr, 15‐cm double‐pigtail plastic stent (Through & Pass Double Pigtail; Gadelius Medical KK, Tokyo, Japan) was placed from the stomach to the cyst. Subsequent ERCP demonstrated resolution of the biliary compression; therefore, the transpapillary biliary stent was removed. The patient was subsequently discharged in good condition.

Twenty‐two days after discharge, the cystic infection recurred due to stent occlusion (Figure 2c). Combined internal and external drainage was performed. One week later, three 7‐Fr double‐pigtail plastic stents (Through & Pass Double Pigtail) were placed for internal drainage after dilation of the ESCR with an 8‐mm balloon catheter (REN biliary balloon catheter, KANEKA, Osaka, Japan). The hepatic cyst collapsed (Figure 2d), and the patient had no further infection recurrence. The indwelling stent has remained in place without exchange for 10 months to date.

## 3 Discussion

Hepatic cyst infection can occur via several routes. In this case, although ERCP did not demonstrate any definite communication, cyst infection developed after conversion from ENBD to transpapillary stenting, and the culture grew Klebsiella, suggesting an intestinal source. Therefore, retrograde infection of the hepatic cyst via the biliary stent and an occult micro‐communication may have occurred [8].

PCD and surgical intervention are standard treatments for infected hepatic cysts [9]. However, in this case, surgical fenestration was contraindicated by the patient's poor general condition, and PCD was considered high risk because cyst‐induced compression of adjacent vessels, the bile ducts, and the gallbladder increased the likelihood of vascular or biliary injury. Furthermore, unpredictable cyst collapse after drainage could have led to stent migration, and the catheter could not be removed if continuous fluid discharge persisted.

Thus, EUS‐CD is a safer and more controlled alternative [1, 2, 3, 4, 5, 6, 7]. Transgastric puncture can prevent vascular and bile duct injuries, and a long drainage tube allows secure positioning within the cyst cavity. Although direct cyst puncture carries the risk of peritonitis, these complications can be prevented by avoiding tract dilation and aspiration of cyst contents using a drainage tube.

In this case, a single external drainage was selected as the initial procedure to prevent peritonitis after tract dilation and to minimize the risk of stent migration after cyst shrinkage. The simultaneous placement of internal and external drainage was avoided because it could have caused leakage of the cystic fluid through the gaps between the stents. Furthermore, the cyst fluid was highly viscous and prone to recurrent obstruction, requiring frequent irrigation. Therefore, external drainage alone was considered the most appropriate initial treatment option.

Infected hepatic cysts differ from liver abscesses in that continuous cystic fluid production complicates infection control and increases the risk of recurrence, often necessitating prolonged or repeated drainage. Surgical fenestration and sclerotherapy with ethanol or minocycline are effective in reducing the recurrence of large symptomatic hepatic cysts [9]. A recent study showed good long‐term outcomes with ethanol retention therapy delivered via both percutaneous and EUS‐guided approaches [10]. In the present case, after infection was controlled by EUS‐guided nasocystic drainage, surgical fenestration was chosen as definitive therapy, but was not feasible because the cyst had already collapsed and the wall remained inflamed. Sclerotherapy was also avoided because, given the patient's postoperative deterioration, another invasive procedure was considered undesirable. A small retrospective study comparing EUS‐guided drainage with 7‐Fr plastic stents and PCD plus minocycline sclerotherapy in symptomatic hepatic cysts reported greater cyst reduction, shorter hospitalization, and comparable recurrence with EUS‐CD [7].

Based on these considerations, internal stenting was performed after intraoperative confirmation that the ESCR tract had matured. Although infection recurred after the first internalization, long‐term resolution was ultimately achieved by placement of multiple plastic stents via ESCR; the cyst collapsed, and the patient has remained free of recurrent infection for 10 months without stent exchange. Multiple stents may have provided sustained drainage through the inter‐stent spaces, contributing to durable cyst collapse.

In conclusion, a stepwise EUS‐CD approach, starting with external drainage and followed by internalization with multiple stents, can be a safe and effective minimally invasive strategy for giant infected hepatic cysts.

## Author Contributions

**Kazuki Endo** drafted and prepared the manuscript. **Haruo Miwa**, **Shotaro Tsunoda**, **Akihiro Funaoka**, **Ritsuko Oishi**, **Yuichi Suzuki**, **Yusuke Takeshita**, **Tomoaki Takahashi**, **Manabu Morimoto**, and **Shin Maeda** contributed to revising the manuscript. All authors have read and agreed to the published version of the manuscript.

## Funding

The author has nothing to report.

## Conflicts of Interest

The authors declare no conflicts of interest.

## Ethics Statement

Institutional Review Board approval was not required for this case report.

## Consent

Informed consent was obtained from the patient for publication of this case report.

## Clinical Trial Registration

N/A
